# Access to an mHealth Tool for Symptom Management in Pediatric Oncology Care: Triangulation Study

**DOI:** 10.2196/93934

**Published:** 2026-07-02

**Authors:** Angelica Höök, Emma Forsgren, Maria Björk, Charlotte Castor, Emma Nordh, Stefan Nilsson

**Affiliations:** 1 Institute of Health and Care Sciences, Sahlgrenska Academy, University of Gothenburg, Box 457, Göteborg, Västra Götaland, 40530, Sweden, 46 736855342; 2 University of Gothenburg Centre for Person-Centred Care, Sahlgrenska Academy, University of Gothenburg, Göteborg, Västra Götaland, Sweden; 3 Crown Princess Victoria Children´s Hospital, Region Östergötland, Linköping, Östergötland, Sweden; 4 The CHILD Research Group, Department of Nursing, School of Health and Welfare, Jönköping University, Jönköping, Sweden; 5 Department of Health Sciences, Faculty of Medicine, Lund University, Lund, Sweden; 6 The Institute for Palliative Care, Region Skåne, Lund University, Lund, Sweden; 7 Department of Biomedical and Clinical Sciences, Linköping University, Linköping, Sweden; 8 Queen Silvia Children´s Hospital, Sahlgrenska University Hospital, Göteborg, Sweden

**Keywords:** pediatric oncology, digital tool, access, symptom assessment, mHealth, PicPecc, Electronic Faces Thermometer Scale, eFTS, mobile health

## Abstract

**Background:**

Digital health offers opportunities to facilitate symptom assessments and communication for children with cancer, particularly after discharge. However, access to these tools must be established to ensure that they effectively support the user. PicPecc (Pictorial Support in Person-Centered Care for Children) is a mobile health tool developed to enable children to remotely assess symptoms and communicate with health care professionals. Understanding access to PicPecc is essential for evaluating its use in pediatric oncology.

**Objective:**

The aim was to test a digital intervention with PicPecc in pediatric oncology care through the lens of access to technology.

**Methods:**

This study uses a triangulation approach to determine access to digital technology through an intervention, PicPecc outside hospital. Fourteen children (6‐17 y), 5 parents, and 6 nurses from 2 pediatric oncology units in Sweden participated. Children were encouraged to use PicPecc for 2 weeks (achieving a median of 14, IQR 9.75-16 days) following hospital discharge to assess pain, nausea, sleep disturbances, and feelings using an assessment scale, pictures, personal notes, and a chat function. Nurses monitored assessments and responded via the administrative interface. Access was analyzed through interviews and an instrument, and by recording the consumption of PicPecc. Data analysis was based on the 5 dimensions of access (availability, accessibility, accommodation, affordability, and acceptability).

**Results:**

The intervention, PicPecc outside hospital, supported availability by enabling children to communicate symptoms in a safe and structured way. Children and parents mentioned feeling safe when they were discharged from the hospital, and nurses perceived it as a valuable complement to follow-up after discharge. PicPecc outside hospital was generally accessible, although initial challenges with log-in procedures related to the PIN code were common. Barriers related to accommodation included interpreting the scale and obtaining an overview of assessments. Affordability was high, as internet access and device availability were not barriers; however, children’s motivation varied depending on symptom burden. Acceptability was strong among children up to 12 years of age, who appreciated the design and gaming function, while the older children found the visual design less age-appropriate.

**Conclusions:**

Access to the mobile health tool, PicPecc outside hospital, appears promising for supporting remote symptom assessment in pediatric oncology, particularly among children up to 12 years of age. However, identified barriers, such as motivational factors and integration into the health care system, need to be addressed.

## Introduction

### Person-Centered Care and Digital Technology

Children (0‐17 years of age) have the right to express their opinions in matters affecting them, including decisions about their health care [1]. To realize this, they need to be provided with opportunities to be heard and to communicate their experiences, preferences, and needs [2].

Person-centered care, according to the model developed by the Gothenburg Centre for Person-Centered Care, involves shared decision-making between 2 experts. The child, together with their parents, is an expert on their lifestyle, preferences, beliefs, values, and health issues, whereas the health care professionals are experts on the management of the disease [3]. Such shared decision-making requires that the child is listened to and supported in communicating their own perspectives. Within pediatric oncology, a person-centered approach to care requires attention to both the physiological side effects of treatment and the subjective experience of symptoms. Assessment strategies should prioritize self-reports whenever possible, considering children’s developmental stage and individual preferences for expressing their symptom experiences [4]. According to Withycombe et al [5], the core symptoms during childhood cancer therapy are anxiety, fatigue, nausea and vomiting, pain, sadness/depression, and sleep disturbances. These symptoms, apart from vomiting, are subjective, and self-reporting is recommended [5].

Digital technology is already a significant part of children’s daily lives, as they regularly use mobile devices. Providing children with validated tools for symptom self-assessment may enhance their ability to communicate how symptoms affect them. Children may also prefer digital tools for nonverbal communication regarding symptoms [6].

The use of digital health technologies can also extend symptom assessments beyond hospital settings, enabling children to independently access symptom assessments as a complement to standard care [7]. Early integration of strategies for symptom management may minimize physical and psychosocial distress and reinforce the expectation that symptoms can be managed. Health care professionals may support children and their families in developing and applying symptom self-management strategies to use at home [4].

**Figure 1.**
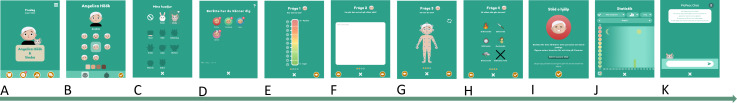
User interfaces of the mobile health tool, PicPecc (Pictorial Support in Person-Centered Care for Children). (A, B) Avatar creation interface for selecting skin color, hair color, hairstyle, and facial expressions. (C) Gaming function rewarding virtual pets for completed symptom assessments. (D) Menu for selecting a specific symptom to assess. (E) Electronic Faces Thermometer Scale (eFTS) for rating symptom severity. (F) Text interface for writing personal notes about symptoms. (G) Interactive body map for identifying symptom locations. (H) Image-based selector for describing the nature of a symptom. (I) External link directing users to symptom management videos on YouTube. (J) Graphical overview for reviewing historical symptom rating data. (K) Chat interface for asynchronous communication with health care professionals.

A mobile app, PicPecc (Pictorial Support in Person-Centered Care for Children), was developed within children’s health care to facilitate remote symptom assessment [8]. In PicPecc (Figure 1), children can create an avatar by selecting skin color, hair color, hairstyle, and facial expression. A gaming function encourages children to assess symptoms by rewarding them with virtual pets for completed assessments. Ratings for symptoms are given by choosing the symptom to be assessed and rating its severity on the electronic Faces Thermometer Scale (eFTS). Users can write personal notes, indicate the location of a symptom on a body map, and use images to describe the nature of a symptom. After rating symptoms, a link is available that directs users to YouTube, where videos on symptom management can be accessed. Users can view their previous symptom ratings in a graph, and the app features a chat function for asynchronous communication with health care professionals (Figure 1).

In remote health care, access is crucial and is proposed as the “fit” between the provider and the client [9]. Access should be viewed as an overarching concept describing how the needs of patients align with the capabilities of health care. Five dimensions of access are proposed: availability, accessibility, accommodation, affordability, and acceptability [10]. These dimensions have been further developed by Sieck et al [9], who state that the technology functions as a mediator between the patient and health care, to which both need access. To utilize the benefits of digital health technology for symptom assessment and management, access to the technology needs to be tested.

### Aim

The aim was to test a digital intervention with PicPecc in pediatric oncology care through the lens of access to technology.

The research questions are as follows:

- How available is PicPecc outside hospital to support the participants’ needs and abilities?
- How accessible is PicPecc outside hospital in relation to participants’ digital skills and literacy?
- How does PicPecc outside hospital accommodate (or adjust to) participants’ ability to navigate it?
- How affordable is PicPecc outside hospital in relation to participants’ money, time, and energy?
- How willing are the participants (acceptability) to use PicPecc outside hospital?

## Methods

### Design

This study employed a triangulation approach based on qualitative interviews, a quantitative instrument, and consumption data to determine access to digital technology through an intervention, PicPecc outside hospital.

### Setting

Participants were recruited from 2 pediatric oncology centers in Sweden where children aged 0 to 17 years are treated for various forms of childhood cancer. The most common inpatient oncology treatments (apart from the treatment of side effects) are chemotherapy and surgery. After discharge from inpatient care, follow-up care varies depending on the treatment and diagnosis. Children who have received chemotherapy are often monitored with blood tests, either at a primary care center, a local hospital, or by home-based health care. If the child or the parents have questions, they are encouraged to contact the pediatric oncology center or the local hospital by phone. Postoperative follow-up also varies, depending on the diagnosis and type of surgery. Children with a diagnosis that requires additional treatment will return to the hospital as soon as they are ready to receive this treatment. For children undergoing surgery as the sole treatment, without additional chemotherapy or radiation, the child and the family return to the hospital for follow-up approximately 2 to 6 weeks after discharge.

### Participants

#### Children and Parents

A convenience sampling strategy was used. Inclusion criteria were children who had started treatment with chemotherapy or surgery and were scheduled for discharge. A variety of ages and diagnoses was an additional criterion. The parents of participating children were also invited to participate. Participants needed to be able to understand and communicate in Swedish or English and have access to the internet at home. Exclusion criteria were children who were unable to participate in data collection themselves.

A total of 14 children were included (median age 14, IQR 7.75-15.25 years), 6 of whom were female. They represented a variety of ages and diagnoses, including leukemia and lymphomas, tumor cerebri, and solid tumors. The time from diagnosis to the start of symptom assessment with PicPecc outside hospital ranged from 2 weeks to more than 3 years. Children under the age of 15 years were informed about the study together with their parent(s) and then decided jointly whether they would participate dyadically or if the child would participate independently. Regardless of whether children participated together with their parent, PicPecc was downloaded to one device with one login. Five parents were included as part of the intervention and used PicPecc outside hospital together with their child. One of the parents and their child declined participation in the interview due to a lack of time. Characteristics of the participating children are shown in Table 1.

**Table 1.** Characteristics of participating children.

Participant	Age (y)	Diagnosis	Time since diagnosis (mo)	Parent participating in the intervention
1	17	Solid tumor	6	
2	16	Tumor cerebri	1	
3	6	Leukemia/lymphoma	3	✓
4	6	Leukemia/lymphoma	2.5	✓
5	8	Tumor cerebri	0.5	✓
6	11	Tumor cerebri	1	
7	14	Solid tumor	2	
8	7	Tumor cerebri	0.5	✓
9	12	Leukemia/lymphoma	4	
10	14	Solid tumor	6	
11	16	Solid tumor	40	
12	11	Leukemia/lymphoma	4	✓
13	15	Leukemia/lymphoma	2	
14	17	Leukemia/lymphoma	2.5	

#### Health Care Professionals

A purposive sampling strategy was used. The inclusion criteria were experienced health care professionals who were able to give advice to participating children and parents and were working in the inpatient and/or outpatient unit at the pediatric oncology center. Participants had to be able to understand and communicate in Swedish or English. A total of 6 nurses were included, whose experience in pediatric oncology ranges from 5 to 25 years. At one site, 3 nurses in the inpatient unit were responsible for including the children and giving them access to PicPecc, while a nurse in the outpatient unit responded to assessments and chats. At the other site, 1 nurse handled everything: including the children, giving access to PicPecc, and responding to assessments and chats. Characteristics of all participants are described in Multimedia Appendix 1.

### Intervention

**Figure 2.**
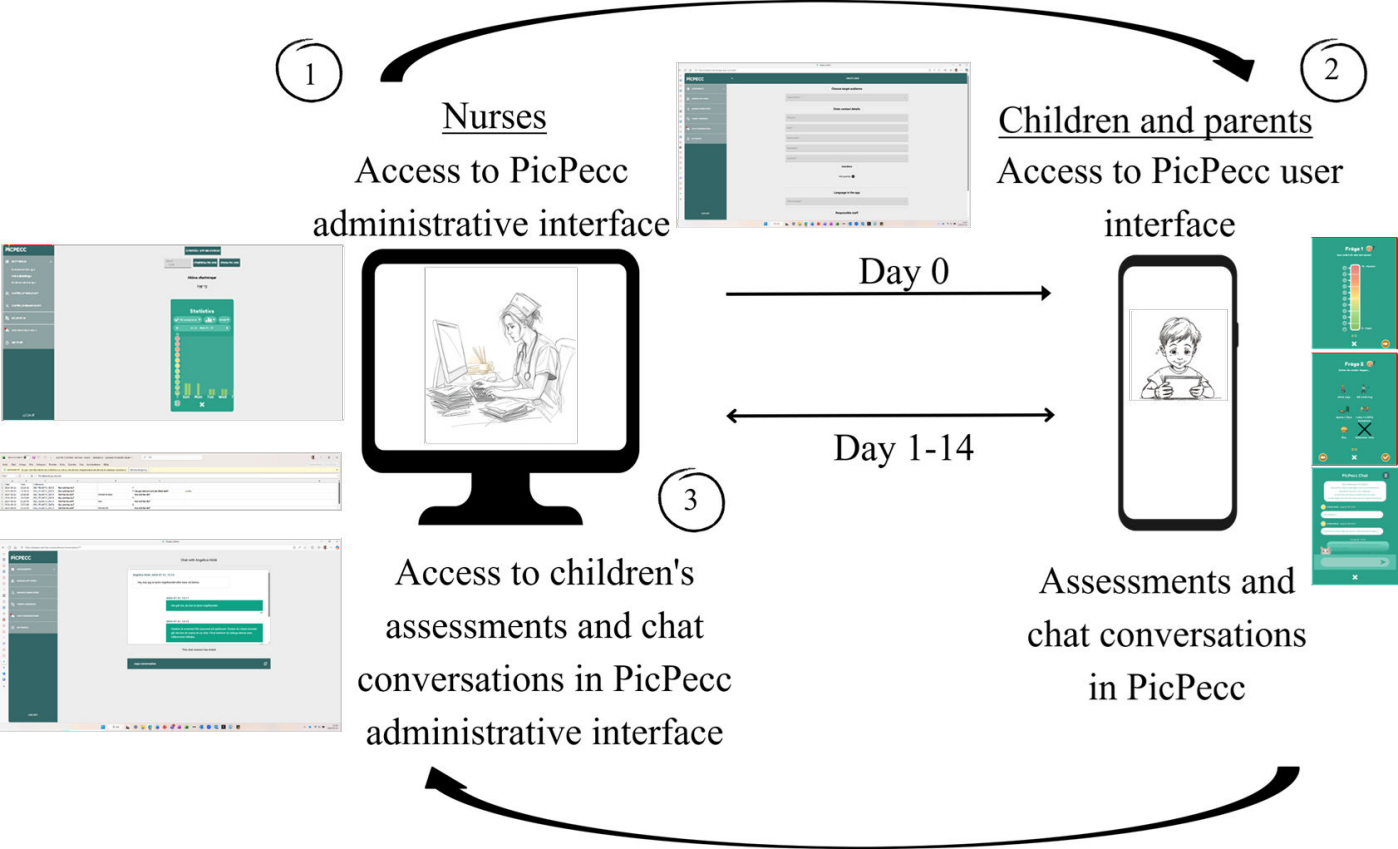
Description of the intervention PicPecc (Pictorial Support in Person-Centered Care for Children) outside hospital.

The intervention (PicPecc outside hospital) consists of 3 steps (Figure 2).

1. Access to PicPecc administrative interface for nurses to create a user. During inpatient treatment, a participating nurse at each center helped the participating children to download PicPecc to perform symptom assessments. A personal code ensured that their assessments remained anonymous to the researchers.
2. Access to PicPecc app for children. Both the child and the parent had the opportunity to review and test the app before discharge. After discharge, participants were encouraged to complete symptom assessments at least twice daily for one or more symptoms over a period of 2 weeks or until the next inpatient hospital stay, whichever occurred first.
3. Nurses’ access to children’s assessments and chat conversations as well as their responses to assessments and chat conversations.

### Data Collection

Data were collected from participating children, parents, and nurses between September 2024 and June 2025.

#### Interviews

Interviews were semistructured, with an interview guide based on the 5 “A”s of access for techquity: availability, accessibility, accommodation, affordability, and acceptability [9]. The same interview guide was used for children and parents, while a modified interview guide with less focus on the PicPecc user interface was used when interviewing nurses (see MultimediaAppendices 2  3). The first author (AH) conducted the interviews in person (8 interviews) or digitally via Zoom (11 interviews). Interviews were audio recorded in a separate unit. Children with participating parents were interviewed dyadically.

#### Instrument

Usability may be seen as one component of access, influencing how users interact with and benefit from a digital tool. The validated instrument, System Usability Scale (SUS), is used for evaluating the usability of digital systems among adults. This instrument was published by Brooke [11] including 10 items formulated as statements rated on a 5-point Likert scale ranging from strongly disagree to strongly agree. SUS has been psychometrically tested for adults in a Swedish context and has demonstrated suitability for evaluating digital tools [12].

In this study, an adapted and more child-friendly version of the instrument, System Usability Scale—Child (SUS-C), was used, including shorter sentences and emojis to clarify response options.

SUS-C was completed after participants had used PicPecc, before the interviews took place. The same version (SUS-C) of the instrument was used for all participants, regardless of age.

#### Consumption

Children and parents included in the study were asked to assess the categories such as *pain*, *nausea*, *sleep disturbances*, and *feelings*—symptoms identified as core symptoms within pediatric oncology [5]. All categories used the eFTS for the assessment of intensity [13]. *Pain* also used a body map, images describing the nature of pain, and an option to add personal notes. *Nausea* included the option of writing personal notes. The category *sleep* used images describing how tiredness affected the child during the day. The category *feelings* used personal notes and images illustrating different feelings (angry, disappointed, tired, sad, happy, and none of the above).

The chat function was used as agreed upon by the participants, with regular responses based on assessments and communication through the chat when needed.

Participating nurses were encouraged to log in twice a day to the PicPecc administrative interface. The frequency of these checks was determined in consultation with the participating children and their parents.

The number of assessments and chat conversations was retrieved from the PicPecc administrative interface and the protocol where nurses documented their monitored assessments and chat conversations.

### Data Analysis

The 5 dimensions of access defined by Sieck et al [9] were adapted into a framework for data analysis through 4 phases inspired by the action research spiral [14]. The authors represent different professional backgrounds with extensive experience in pediatric health care and have previously worked with the concept of access.

In the initial phase, 2 of the authors (AH and EF) translated the dimensions and established a preliminary framework. The second phase included 5 of the 6 authors (AH, EF, MB, CC, and SN) to explore the framework through collective discussions. The third phase involved understanding and interpreting how these dimensions could be used during the analysis. During the final phase, there was a collaborative refinement and clarification of the dimensions and the different components (interview, instrument, and consumption), which resulted in the final framework, presented as Table 2.

**Table 2.** Description and interpretation of the 5 dimensions of access “A”s.

Dimension	Definition by Sieck et al [9]	Research questions	Data collection
Availability	The relationship between existing telehealth services provided by a system and resources to the patient’s need and ability	How available is PicPecc^a^ outside hospital to support the participants’ needs and abilities?	Interview Item 9: *9. I felt very confident using the system*. Number and content of chat conversations
Accessibility	The relationship between digital skills and literacy of a patient population and the support available to use them	How accessible is PicPecc outside hospital in relation to participants’ digital skills and literacy?	Interview Items 4, 7, and 10: *4. I think that I would need the support of a technical person to be able to use this system*. *7. I would imagine that most people would learn to use this system very quickly*. *10. I need to learn a lot of things before I could get going with this system*.
Accommodation	The relationship between requirements of digital platforms and the patient’s ability to navigate them	How does PicPecc outside hospital accommodate (or adjust to) participants’ ability to navigate it?	Interview Items 5, 6, and 8: *5. I found the various functions in this system were well integrated*. *6. I thought it was too much inconsistency in this system*. *8. I found the system very cumbersome to use*. Number of assessments regarding different ways to assess symptoms
Affordability	The relationship between the costs of internet services and devices and the patient’s ability to pay for them	How affordable is PicPecc outside hospital in relation to participants’ money, time, and energy?	Interview Items 2 and 3: *2. I found the system unnecessarily complex*. *3. I thought the system was easy to use*. Number of assessments for different symptoms
Acceptability	The relationship between the health care organizations, telehealth tools, and workflows and the patient’s attitude toward and comfort with tools and workflows	How willing are the participants (acceptability) to use PicPecc outside hospital?	Interview Item 1: *1. I think that I would like to use this system frequently*. Assessments overall and number of days

a PicPecc: Pictorial Support in Person-Centered Care for Children.

The dimension availability, focusing on participants’ needs and abilities, was reflected in the SUS-C item addressing confidence (item 9), as well as in the use of the chat function. Accessibility, which relates to digital skills, was reflected in SUS-C items concerning the need for technical assistance and the need for digital competence. Accommodation was reflected in SUS-C items regarding how the system, as well as its individual components, functioned together and how these components were used. Affordability was reflected in SUS-C items addressing the perceived complexity of the system, as well as in the extent to which different symptoms were assessed. PicPecc, as an application, was free to download and use. Finally, the dimension acceptability was reflected in the frequency of use in both SUS-C and consumption.

### Qualitative Analysis

Interview duration ranged from 7 to 27 minutes (median 16.44, IQR 11.92-22.16 minutes). Data were analyzed by the first and second authors (AH and EF) in NVivo 15 (Lumivero) using the template analysis [15]. AH is a pediatric nurse and a PhD student, and EF is a speech and language therapist with a PhD degree. Coding was primarily deductive, guided by *a priori* themes: availability, accessibility, accommodation, affordability, and acceptability. The initial template was based on these 5 themes. During the coding process, AH and EF independently identified codes and assigned them to the *a priori* themes. The coding was then compared and discussed, and any discrepancies were resolved through discussion until consensus was reached between AH and EF. In the following step, subthemes were developed by grouping codes related to the same dimension, and this was also conducted independently by AH and EF. Differences in interpretation were addressed through discussions (also involving SN, CC, and MB) until agreement was reached. The final template is structured hierarchically, with the *a priori* themes as main themes and subthemes identified in the transcribed interviews [15]. A table summarizing the themes and subthemes is provided in Multimedia Appendix 4, and subthemes are exemplified by citations from participants.

### Statistical Analysis

#### Instrument

When analyzing each item in SUS-C, scores ranged from 0 (strongly disagree) to 4 (strongly agree).

The total score was calculated using the sum for each item, contributing to a value between 0 and 4. For items 1, 3, 5, 7, and 9, the score was equal to the scale position. For items 2, 4, 6, 8, and 10, the score was equal to 4 minus the scale position. The total sum is then multiplied by 2.5 to obtain the overall score, ranging from 0 to 100 [11], where a higher number represents a higher usability [11,16] (see Multimedia Appendix 5).

Descriptive statistics, including median, range, and frequencies (%), were calculated for single items, as well as for the total score, using IBM SPSS version 30.0 (see Multimedia Appendix 6).

#### Consumption

Descriptive statistics, including median, mean, and frequencies (%), were calculated for demographic variables and assessments using SPSS version 30.0.

### Ethical Considerations

This study was approved by the regional ethical review board (Dnr 2024-02450-01). Informed written consent was obtained from the accompanying parent of the children aged 6 to 14 years and directly from participating children aged 15 to 17 years. Assents were obtained from children aged 6 to 14 years. The children were informed that both parental and child assents were required. Assent forms for children were designed for different age groups and included an assent form based on pictures.

## Results

### How Available Is PicPecc Outside Hospital to Support Participants’ Needs and Abilities?

#### Qualitative Findings

##### Interviews

Different perceptions of availability were further elaborated in the interviews and are presented as subthemes as follows.

##### Supporting Dialogue and Acting as a Link

The analysis showed that PicPecc functioned as a facilitating tool in children’s everyday lives, supporting both symptom reporting and communication between home and health care. Children and parents described PicPecc as easy to use in their daily lives together and as a practical way to express how the child felt without face-to-face communication. The children were able to communicate symptoms in a way that felt safe and simple, and symptoms arising at home were monitored remotely.

*There is some sense of security in knowing that it is still being followed up even when you are at home, that it does not just get forgotten and you only have to call if something happens. Instead, you can check in every day and know that someone is keeping track of it*

[Parent to a 6-year-old]

##### Encouraging Involvement

Children appreciated being more involved in their care when using PicPecc outside hospital. The reporting of symptoms made children pay attention to how they felt, as well as take an active role in communicating their needs. Children mentioned that PicPecc served as a place to “get things off their chest” and to express emotions that were difficult to express verbally. Reflecting on their experiences was another aspect that made PicPecc outside hospital useful.

*Maybe it’s a bit easier than when someone comes in and asks you directly. Here, you can just sit and think for yourself, without someone standing there watching you and asking questions*

[14-year-old]

##### Facilitating Reflection and Gaining an Overview

An overview of symptoms was mentioned as an important aspect of PicPecc outside hospital. The overview acted as a heads-up for nurses, as well as a reminder for the children regarding how their last few days had been. Both children and nurses appreciated being able to observe changes in symptom experiences.

##### Offering a Parent Version

Both parents and nurses mentioned the value of a parent version (which is not available today), where parents could have their own login, obtain information, and ask questions directly. Nurses noted that parents have informational needs that are not addressed through the child’s login.

*It just occurred to me now, if, as a parent, you would want to ask questions. Because this is his tool, but if you are worried as a parent and want some support or reassurance*

[Parent to an 11-year-old]

### Quantitative Findings

#### Instrument

Participants reported feeling confident using the system, SUS-C item 9 (Table 3).

**Table 3.** System Usability Scale—Child (SUS-C) divided into positive and negative items with participants’ median values^a^.

Items	Statement	Score (range 0-4), median (IQR)
Positive-related items		
1	I think that I would use this system frequently.	2 (2-3)
3	I thought the system was easy to use.	4 (4-4)
5	I found the various functions in this system were well integrated.	3 (2-4)
7	I would imagine that most people would learn to use this system very quickly.	4 (3-4)
9	I felt very confident using the system.	4 (3-4)
Negative-related items		
2	I found the system unnecessarily complex.	0 (0-0)
4	I think that I would need the support of a technical person to be able to use this system.	0 (0-0)
6	I thought it was too much inconsistency.	0 (0-1)
8	I found the system very awkward to use.	0 (0-0)
10	I needed to learn a lot of things before I could get going with this system.	0 (0-1)

a 0 equals strongly disagree, and 4 equals strongly agree.

#### Consumption

To further address the question of PicPecc’s availability, the number of chat conversations, as well as their focus, was recorded. In total, there were 11 chats, 7 of which were started by nurses and 4 by children or their parents. Some chats were aimed at prompting and encouraging the use of PicPecc and assessments, while other chats focused on symptom management, upcoming visits to the hospital, or functions within PicPecc.

In summary, several participants expressed feeling more confident at home when they had access to PicPecc, and this was reflected in the instrument, as well as in the interviews. The chat conversations also highlighted availability, as the children had noticed the chat function even though they had not used it.

### How Accessible Is PicPecc Outside Hospital in Relation to Participants’ Digital Skills and Literacy?

#### Qualitative Findings

##### Interviews

Different perceptions of accessibility were further elaborated upon in the interviews and are presented as subthemes as follows.

##### Finding and Downloading

PicPecc outside hospital was described as simple to use, referring to the ease of finding and downloading PicPecc, the support available during the download process if required, and the self-explanatory nature of the content. However, there was no online support for the nurses providing PicPecc to the children.

##### Logging in With a PIN Code

Barriers to managing PicPecc referred to challenges in logging in. These challenges were perceived by all participant groups and occurred when logging in for the first time. The PIN code was also seen as a barrier, as it was difficult to remember and difficult to change. This was more of an issue when the participants tried to use PicPecc for the first few times; however, after logging in a few times or changing the PIN code, participants reported it worked well.

*…and we got used to the numbers; they got ingrained as a muscle memory*

[Parent to a 6-year-old]

*I remember there was a bit of trouble with it. I had to try for a while before it worked. But I think I had the wrong code at first, if I remember correctly*

[16-year-old]

##### Presenting/Offering Technical Support

Children, parents, and nurses all described support as a facilitator, as the available support made technical challenges more manageable. During the data collection period, there was a major problem with the administrative interface, resulting in one child not being able to use the chat function and the nurse being unable to monitor any assessments. This child chose to use PicPecc for a longer period so they would have the opportunity to try all the different features of PicPecc.

### Quantitative Findings

Results from the SUS-C indicated that most (n=18) participants perceived PicPecc outside hospital as easy to learn (item 10), easy to use and manage (item 7), and did not need any support when using it (item 4) (Table 3, Multimedia Appendix 6).

### How Does PicPecc Outside Hospital Accommodate (or Adjust to) Participants’ Ability to Navigate It?

#### Qualitative Findings

##### Interviews

Different perceptions of how well PicPecc accommodated users’ abilities were further elaborated upon in the interviews and are presented as subthemes as follows.

##### Pictorial Support

Children and parents described the app as easy to navigate as they moved between different sections, and the pictures supported their understanding and orientation. However, children who were unable to write needed help from their parents to compose their personal notes. Reminders were an additional function that assisted in identifying unanswered questions.

*I looked through all the sections, and I didn’t find anything difficult to locate or navigate*

[17-year-old]

##### Assessment Scales and Statistics

The assessment scales were sometimes difficult to interpret and caused confusion. For one category, a high number represented positive well-being, while for the other categories, a high number represented negative well-being.

The children and nurses described the statistical view as difficult to interpret, identifying the graphs as difficult to read. They mentioned challenges in obtaining an overview of their assessments for a specific symptom or over a longer period and stated that a clearer statistical overview would be valuable for observing changes over time.

Contextual factors refer to barriers experienced by nurses, for example, the fact that the PicPecc user interface and administrative interface differ and that the nurses felt the administrative interface was less intuitive and more time consuming.

*And then you get an overview, and you can see everything. But you can’t see whether they have… if they have written a comment, unless you click your way into the Excel sheet. And then there’s a lot of clicking and a lot of scrolling, back and forth. And if you have lots of patients at the same time… well, then you can see them all at once there. Yes, but sometimes I feel there’s a bit too much clicking to, well… to get a good overview*.

[Nurse]

### Quantitative Findings

#### Instrument

Of the 23 participants who answered item 5, 9 thought that the various functions in PicPecc outside hospital were not well integrated. However, of the 24 participants who answered item 8, 20 participants answered “strongly disagree” regarding whether they found the system very cumbersome to use (Multimedia Appendix 6).

#### Consumption

Different categories had different ways of describing symptoms (although all categories used eFTS to assess symptom intensity). Pictures were most frequently used in the category *Sleep* and *Feelings*, whereas personal notes were used more frequently in the category *Nausea* (Table 4).

**Table 4.** Number of assessments in different categories divided by ways of describing symptoms.

Ways of describing symptoms	Pain, n	Nausea, n	Sleep, n	Feelings, n
eFTS^a^	296	248	252	271
Body map	32	—^b^	—	—
Personal notes	51	81	—	4
Pictures	85	—	226	228

a eFTS: Electronic Faces Thermometer Scale.

b Not available.

### How Affordable Is PicPecc Outside Hospital in Relation to Participants’ Money, Time, and Energy?

#### Qualitative Findings

##### Interviews

Different perceptions of affordability were further elaborated upon in the interviews and are presented as subthemes as follows.

##### Owning a Device

Some children reported that they used their parents’ device for making assessments, even though they were able to borrow a device from the ward. One parent stated that having PicPecc on their device limited the child’s opportunities to complete assessments.

##### Motivation and Prioritization

Children’s energy, fatigue, and treatment side effects affected their engagement in PicPecc. However, when they were feeling well, assessing symptoms felt less meaningful.

*I was so tired that I didn’t even have the energy to log in*

[14-year-old]

*Yes, I think you used it for about a week or so, every day. Then when you weren’t in pain anymore, you didn’t find it very interesting…*

[Parent to a 7-year-old]

##### Organizational Demands/Requirements

Nurses expressed concerns about who would take responsibility for assessments and chat conversations if PicPecc outside hospital was implemented. The fact that assessments and chats needed to be manually reported in the electronic health care record was regarded as time-consuming.

*Who is supposed to be on the receiving end of it? I think that’s also something that needs to be addressed before using it in real clinical practice*

[Nurse]

### Quantitative Findings

#### Instrument

Almost all participants (n=21) perceived the app as easy to use, according to their answers to items 2 and 3 (Table 3, Multimedia Appendix 6).

#### Consumption

The participants’ use of the different categories was one indicator of how relevant PicPecc outside hospital was to their symptom experience. Assessments in PicPecc were divided into 4 different categories. Pain was assessed 296 times, nausea 248 times, sleep 252 times, and feelings were assessed 271 times (Table 4).

Most children made 21 to 30 assessments in different categories, but some only assessed different categories 0 to 10 times. Nurses logged into the administrative interface 4 to 10 times for each child to observe any completed assessments and chat conversations.

### How Willing Are the Participants (Acceptability) to Use PicPecc Outside Hospital?

#### Qualitative Findings

##### Interviews

Different perceptions of acceptability were further elaborated upon in the interviews and are presented as subthemes as follows.

##### Design Elements in Layout and Colors

Participants perceived the visual design of PicPecc as colorful and appealing. The colors were described as intuitive, nice, or fun, which contributed to a positive experience. However, some participants found the colors to be contradictory to the text.

Some older children (13‐17 years of age) thought the design was somewhat childish. Nurses described how they declined to participate when they saw the instructions because the design did not appeal to them.

*The six-year-olds and the younger ones up to around eleven thought it was great. But the older boys… if you asked them, they said no immediately, just from seeing the instructions. Sometimes I brought the poster with me, and they could scan the QR code. And then it was like, “No,” straight away*.

[Nurse]

##### Personalization Through the Avatar and Virtual Pets

Personalizing an avatar was experienced as fun, particularly among the younger children (6‐12 years of age). They appreciated having the option to adjust hair color, hairstyle, and skin tone. However, some participants felt that the function was limited, as it lacked options for clothing and hairstyles. The avatar was only used during the initial setup, and some participants found it difficult to change the name of the avatar. The virtual pets, gained after fulfilling assessments, were named, which contributed to personalization.

*It was fun to create your own avatar. And it was fun that you got pets. Those two things were some of the best parts, I think*.

[12-year-old]

##### Personal Engagement

For younger children (6‐12 years of age), virtual pets were seen as a motivating factor, and the reward system created a purpose for completing assessments. However, older children thought the reward system was too childish and did not motivate them to use the app, which was also the opinion of parents and nurses.

Nurses thought the app had potential as a communication tool that could be promising if integrated into health care. Participants had suggestions for dealing with the “childish” aspects of the app, for example, by creating versions suitable for different age groups.

*I don’t think that, at my age, you really look for any kind of reward just for completing the ratings.*

[17-year-old]

### Quantitative Findings

#### Instrument

For item 1, four children answered “strongly agree” to wanting to use PicPecc frequently.

#### Consumption

Collectively, the participating children made 1067 assessments. They started using PicPecc 0 to 4 days after their discharge from hospital and used it for 1 to 27 days, with a median of 14 (IQR 9.75-16) days. Most of the children (n=10) used PicPecc for 11 to 20 days, while 3 children used PicPecc for less than 10 days and 1 child for more than 21 days.

### Summary of Findings

**Figure 3.**
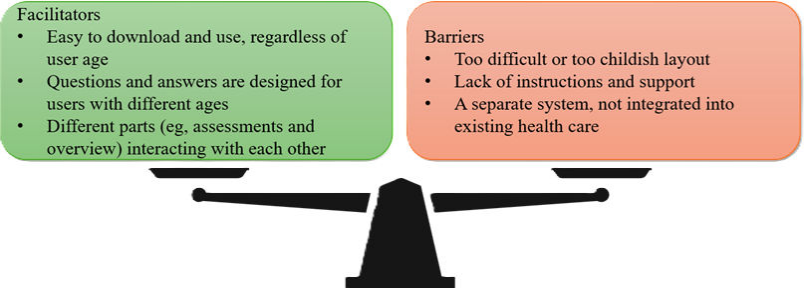
Facilitators and barriers for use of a mobile health tool for remote symptom assessment within pediatric oncology.

A summary of identified facilitators and barriers to a remote symptom assessment is shown in Figure 3.

The triangulation of data obtained from interviews, the instrument, and consumption data confirmed and diverged in findings across the different dimensions of access. Interviews indicated that participants generally appreciated “PicPecc outside hospital,” although the level of consumption varied. Scores from the instrument supported usability.

The sum of median scores for the instrument was high (Table 5), which may indicate that participants found PicPecc outside hospital to have good usability [16]. Scores for each item corresponding to children, parents, and nurses, respectively, are shown in Multimedia Appendix 6.

**Table 5.** Median scores for System Usability Scale—Child (SUS-C).

Participants	Participants, n	Score, median (IQR)
Children	14	86.25 (75-90.62)
Parents	5	82.50 (71.25-97.5)
Health care professional	6	93.75 (85-92.62)

## Discussion

### Principal Findings

The aim was to test a digital intervention with PicPecc (PicPecc outside hospital) in pediatric oncology care through the lens of access to technology. The research questions were related to the dimensions of access [9] and explored PicPecc outside hospital to get an understanding of how mobile health (mHealth) can support children with an oncology diagnosis and their parents when they are at home. The use of triangulation, comparing qualitative and quantitative data, made it possible to explore the perceived and actual use of PicPecc, thereby providing a broader understanding of access. This triangulation identified both convergence and divergence between the collected data.

The overall findings indicated that PicPecc outside hospital was perceived as usable and meaningful and may enable communication regarding symptoms. In combination, the interviews, instruments, and consumption of PicPecc showed that PicPecc outside hospital may facilitate symptom assessments and symptom management and that participants perceived they could access PicPecc outside hospital for symptom assessments and management.

However, the needs and motivational factors among older children, along with challenges related to nurses managing assessments within the health care system, need to be considered. These findings align with the scoping review by Simon et al [17], which found that children were likely to accept new technology and that barriers were mostly related to organizational factors.

Children commented positively on the ability to express their symptoms without face-to-face communication. This, along with the chat conversations, aligns with partnership and shared decision-making in person-centered care and provides children with an opportunity to express how different symptoms affect their daily lives. The chat function was appreciated although not used by everyone. Nevertheless, previous studies have shown that real-time communication functions may facilitate an improved quality of life among adult patients [17]. Some of the children in our study thought that chat conversations were an easy way to get in touch with health care and that they could start a conversation themselves, without involving their parents. Symptom assessment and management are not sufficiently addressed in clinical practice, and providing an opportunity for a real-time conversation is an important part of an mHealth tool [18].

Self-reporting is the gold standard for subjective symptom assessments, and proxy reports do not always align with the experience of the child. Tomlinson et al [19] have shown a significant difference between self-report and proxy report regarding some symptoms. These discrepancies may reflect differences in how children and parents perceive, experience, and interpret symptoms [19]. Using PicPecc together may enhance communication and facilitate the sharing of understandings and needs [7].

PicPecc was described as easy to download, learn, and use. Initially, challenges with the login procedure posed a barrier. This barrier decreased over time and was managed with support from parents or nurses, highlighting the necessity of available assistance. Having your own device may be a prerequisite for using PicPecc. During the data collection, a tablet was made available for children to borrow. However, they all used their own device or their parents’ device. The use of PicPecc may offer new ways of communicating within the family, as well as with health care professionals, and plays an important role in managing symptoms [20,21].

The pictorial support in PicPecc facilitated the understanding of assessments. However, participants reported inconsistencies in how to interpret eFTS across the different categories, as well as difficulties in accessing an overview. Participating nurses also experienced the administrative interface as less intuitive. Previous publications regarding the eFTS used in PicPecc have shown content and construct validity for pain assessments [13,22]. This study revealed no difficulties in using eFTS when 10 represented a negative feeling, but when 10 represented a positive feeling, one child found it confusing. This highlights the need for a supportive function, as well as an introduction to using the different parts of the digital tool.

Apart from *pain*, the category *feelings* was assessed on the greatest number of days and on the largest number of occasions, aligning with findings by Christianson et al [23] who addressed a gap in emotional needs during conversations regarding prognostic discussions within pediatric oncology. PicPecc may act as a tool for discussions regarding emotional needs.

The relationship between the users’ resources in terms of time, money, and energy and the requirements of PicPecc outside hospital identified facilitators and barriers. Financial resources were not seen as a barrier. However, children’s energy levels and symptom burden influenced their motivation to use PicPecc. On the other hand, when symptoms started to diminish, engagement started to fluctuate. The relevance of PicPecc was closely tied to symptom experiences.

In this study, contextual factors were one of the identified barriers, indicating that someone at the hospital needs to be responsible for responding to the chat conversations and monitoring completed assessments. This barrier has also been identified by Simon et al [17], along with financial resources and time as the most common barriers. Symptom management depends on effective communication regarding the participants’ experiences [24,25].

In this study, the younger children (6‐12 years of age) were highly engaged and appreciated the option of customizing an avatar, along with the gaming function of collecting virtual pets. In contrast, the older children (13‐17 years of age) thought that the design was too childish, which made them less willing to participate. The review published by Cheng et al [21] showed that younger children were more involved, and developmental stages were factors that interfered with the interventions. This highlights the necessity of different versions of the tool for different ages or developmental stages, which was also suggested by our participants.

According to the definition of person-centered care, as described by the Gothenburg Centre for Person-Centred Care, a partnership includes shared decision-making. Attitudes toward children’s participation in shared decision-making are gradually changing [3,26]. Using a digital tool where access is secured may facilitate this partnership [27]. This also aligns with the definition of child and family-centered care, in which children are acknowledged to have rights, preferences, and perspectives, and an individualized partnership is achieved through a collaboration between children, parents, and health care professionals [28]. As an mHealth tool equipped with a validated self-assessment scale (eFTS), PicPecc may support children in expressing their views and in becoming partners in their care, aligning with the Convention on the Rights of the Child [1,8].

The credibility and trustworthiness of the qualitative findings were strengthened using a triangulation approach that combined data from interviews, instruments, and consumption. The analysis was performed with a template that included predefined themes based on a framework for access. During the analysis, several subthemes emerged according to participants’ experiences. Two authors with different professional backgrounds were involved in this process, reducing the risk of bias.

### Strengths and Limitations

There are some limitations to this study. First, the interpretation of the 5 dimensions of access was made by the authors, and consequently, aspects of these dimensions may be missing or overlapping. However, the triangulation described access from different perspectives via interviews, ratings using the instrument, and by exploring factual consumption, which could spotlight aspects that one measure alone could have missed. That said, SUS-C has not yet been validated for use in children. In different contexts, adaptations of SUS for use in children have proven that the adapted instrument was feasible and showed good reliability [29,30]. In this study, SUS-C is used as a measure within the triangulation design, and the results from the instrument should be understood as supportive rather than definitive.

The qualitative analysis used a template analysis approach, whereby the 5 dimensions of access acted as *a priori* themes. On the one hand, this made the analysis more structured, but on the other hand, it may constrain the emergence of unanticipated themes, which could have led to overlooking participants’ experiences. However, this was managed during the analysis using subthemes that emerged from participants’ experiences. The use of *a priori* themes may have influenced how data were interpreted and categorized, and regular discussions between the authors were used to ensure that findings were grounded in the data. One of the authors (EF) involved in the analysis had no previous experience in pediatric oncology, which contributed to reducing the influence of context-specific preunderstandings and supported reflexivity.

The study has a rather small sample size, which limits the transferability of the findings. However, the participating children represented a wide range of ages and the most common diagnoses within pediatric oncology.

Participants used PicPecc outside hospital for 1 to 27 days, but for a more thorough experience, they may have to use it for a longer duration. However, children’s engagement with the intervention was influenced by symptom burden and energy levels, which varied during this period. Such variation makes it difficult to determine which barriers are related to the tool itself versus treatment-related factors.

During the study period, there were technical problems that affected the ability to respond to assessments and chat conversations. This issue, along with nurses’ experiences with the administrative interface, highlights the importance of the system’s reliability when further tested.

Finally, some of the participants used their own digital devices for downloading the app, whereas others used their parents’ devices, and this might influence the user experience. Each child having their own device might be necessary to ensure consistent usage among all children at all times of the day.

### Conclusions

Triangulation of qualitative interviews, a quantitative instrument, and consumption through the lens of access showed that PicPecc outside hospital is promising and may benefit children in pediatric oncology care. Our findings indicate that PicPecc may support access to remote symptom management after discharge, particularly for younger children up to 12 years of age. However, access was influenced by several factors; for example, symptom burden and energy levels affected motivation and engagement. Differences in age and development were another factor; children above 13 years of age perceived the design as less relevant. Finally, organizational factors such as responsibility for monitoring assessments and chat conversations, as well as integration into existing health care systems, need to be addressed.

## Supplementary material

10.2196/93934 Multimedia Appendix 1 Participants’ characteristics.

10.2196/93934 Multimedia Appendix 2 Interview guide used for children and parents.

10.2196/93934 Multimedia Appendix 3 Interview guide used for nurses.

10.2196/93934 Multimedia Appendix 4 Themes and subthemes.

10.2196/93934 Multimedia Appendix 5 A guide to calculate the scores for System Usability Scale—Child. 93934-Multimedia-Appendix-5.docx

10.2196/93934 Multimedia Appendix 6 Scores of children, parents, and nurses for each item in System Usability Scale-C.

## Abbreviations

eFTS: electronic Faces Thermometer Scale
mHealth: mobile health
PicPecc: Pictorial Support in Person-Centered Care for Children
SUS: System Usability Scale
SUS-C: System Usability Scale, Child version
