# Supplementary material for: Access to an mHealth Tool for Symptom Management in Pediatric Oncology Care: Triangulation Study
Source: JMIR Form Res. 2026 Jul 2;10:e93934. doi: 10.2196/93934 (PMC13327532; doi:10.2196/93934)
Supplement: Multimedia Appendix 1 [file formative-v10-e93934-s001.docx]

Supplementary file 1, characteristics of participants

| Children (n) | 14 |
| --- | --- |
| Age (md) | 14 (6-17) years |
| Gender |  |
| Female | 6 |
| Male | 8 |
| Time since diagnosis (md) | 2.5 (0.5-40) months |
| Treatment with chemotherapy | 10 |
| Treatment with surgery | 4 |
|  |  |
| Parents (n) | 5 |
| Age (md) | 42 (34-47) years |
| Gender |  |
| Male | 1 |
| Female | 4 |
|  |  |
| Health care professionals (n) | 6 |
| Age (md) | 32 (28-48) years |
| Gender |  |
| Female | 6 |
| Male | 0 |
| Years of experience within paediatric oncology (md) | 9 (5-25) years |
