# Supplementary material for: Access to an mHealth Tool for Symptom Management in Pediatric Oncology Care: Triangulation Study
Source: JMIR Form Res. 2026 Jul 2;10:e93934. doi: 10.2196/93934 (PMC13327532; doi:10.2196/93934)
Supplement: Multimedia Appendix 2 [file formative-v10-e93934-s002.docx]

**Intervjuguide**

Denna intervjuguide kommer att användas för barnen, samma frågor kommer även att användas vid intervju av föräldrar med en viss justering när det gäller hur frågorna ställs.

Hej! Jag heter ….

Vi vill veta vad du tycker om appen PicPecc som du använder/har använt. Det som vi kommer att prata om kommer inte någon annan att ta del av och det du säger kommer inte att kunna spåras tillbaka till dig. Du kan välja att avsluta när du vill och det kommer inte att påverka din vård eller behandling. Det vi pratar om kommer att spelas in och inspelningarna kommer att användas för att lättare komma ihåg vad vi har pratat om. Vi skulle vara tacksamma om du vill hjälpa oss att berätta vad du tycker om appen PicPecc.

**Introduktion / Allmänt**

Den här appen är utvecklad för barn med långvarig sjukdom (cancer och hjärtsjukdom) för att underlätta kommunikation om olika symtom. Barn, föräldrar och vårdpersonal har varit med i utvecklingen. Nu vill vi undersöka hur appen har fungerat när den har använts hemma.

1. Vad tycker du allmänt om appen?
2. Vad tycker du om när det gäller appen?
3. Vad tycker du inte om?
4. Vad tycker du om avatarerna?
5. Vad skulle du ha gjort annorlunda i appen?
6. Är det något som du tycker saknas i appen / något som du skulle vilja lägga till?

**Vad är dina förväntningar (tankar om) appen?**

1. Skulle du vilja ha en app som den här som du kan använda när du vill för att berätta hur du mår? Berätta mer.
2. Tror du att appen kan användas av barn på sjukhus eller hemma? Utveckla, berätta mer.
3. Vad tycker du om djuren som du kan samla när du använder appen? (Skulle du använda appen mer för att kunna samla ytterligare djur?)

**Tillgänglighet (availability)**

1. Kunde du hitta appen på google play eller på appstore? Berätta mer.
2. Hur lätt var det att ladda ner appen? Berätta mer.
3. Hur lätt var det att logga in i appen? Fungerade pinkoden? Berätta mer.
4. Hur lätt var det att hitta i appen? Berätta mer.
5. Vad det möjligt för dig att anpassa avataren för att passa din profil (ändra frisyr, hårfärg, hudfärg mm)? Har du gett din avatar ett namn?
6. Kunde du ändra namnet på ditt husdjur? Har du valt namn på ditt husdjur?

**Prisvärdhet (affordability)**

1. Vad skulle underlätta för dig att använda appen (tillgång till wifi, mobildata, övrigt)?
2. Vad tror du skulle hindra dig från att använda appen? Berätta mer.
3. Kunde du använda länkarna som finns i appen? Berätta mer?

**Godtagbarhet (acceptability)**

1. Vad tror du kan hjälpa dig att använda appen?
2. Vad tycker du kunde ha gjorts annorlunda i appen? Skulle du vilja ändra något i appen, iså fall vad?
3. Vad tycker du om färgerna som används i appen? Tycker du att de fungerar, om inte varför? Är det något du skulle vilja fungerade annorlunda?
4. Hur kunde appen hjälp dig att känna dig bättre?

**Anpassningsbarhet (accomodibility)**

1. Den här appen är framtagen för att hjälpa barn med cancer att kommunicera om olika symtom. Tycker du att appen kan användas av barn för att kommunicera om olika symtom? Berätta mer?

**Sammanfattning/Avslutning**

1. Är det något mer som du vill säga när det gäller appen?

Tack för att du ville hjälpa oss att undersöka hur appen fungerar och hur den kan bli bättre. Du får självklart fortsätta att använda appen hur mycket du vill.
