# Supplementary material for: Access to an mHealth Tool for Symptom Management in Pediatric Oncology Care: Triangulation Study
Source: JMIR Form Res. 2026 Jul 2;10:e93934. doi: 10.2196/93934 (PMC13327532; doi:10.2196/93934)
Supplement: Multimedia Appendix 3 [file formative-v10-e93934-s003.docx]

Intervjuguide vårdpersonal

Bakgrundsdata

Ålder

Kön

Utbildning

Yrke

Erfarenhet

Antal år inom nuvarande yrke

Antal år på nuvarande arbetsplats

PicPecc

Vad tycker du allmänt om appen?

Vad tycker du om när det gäller appen?

Vad tycker du inte om när det gäller appen?

Är det något som du tycker saknas i appen / något som du skulle vilja lägga till?

På vilket sätt tycker du att appen kan användas av barn i hemmet?

Hur lätt var det för barnen att ladda ner appen?

Hur lätt var det att logga in i appen?

Hur lätt var det att logga in i PicPecc Admin?

Hur lätt var det att se barnens skattningar?

Hur lätt var det att svara på chatten?

Vad skulle kunna bli bättre?

Vad skulle kunna underlätta för dig att använda PicPecc admin?

På vilket sätt kan appen användas av barnen att kommunicera om olika symtom?

Åtgärder kopplade till skattningar

Vilken typ av åtgärder har genomförts relaterat till gjorda skattningar?

Vilken typ av åtgärder har genomförts relaterat till chatten?

Vad tycker du fungerade bra i chatten?

Hur skulle vi kunna göra för att göra PicPecc bättre för vårdpersonal att använda?

Tack för att du ville hjälpa oss att undersöka hur appen fungerar och hur den kan bli bättre. Du får självklart fortsätta att använda appen hur mycket du vill.
