# Supplementary material for: Access to an mHealth Tool for Symptom Management in Pediatric Oncology Care: Triangulation Study
Source: JMIR Form Res. 2026 Jul 2;10:e93934. doi: 10.2196/93934 (PMC13327532; doi:10.2196/93934)
Supplement: Multimedia Appendix 4 [file formative-v10-e93934-s004.docx]

Supplementary file 4

The analysis of interviews resulted in the following subthemes related to the *a´priori* themes.

1. Availability

1.1 Supporting dialogue and acting as a link

1.2 Encouraging involvement

1.3 Facilitating reflection and gaining an overview

1.4 Offering a parent version

2. Accessibility

2.1 Finding and downloading

2.2 Logging in with a pin code

2.3 Presenting/offering technical support

3. Accommodation

3.1 Pictorial support

3.2 Assessment scales and statistics

4. Affordability

4.1 Owning a device

4.2 Motivation and prioritization

4.3 Organizational demands / requirements

5. Acceptability

5.1 Design elements in layout and colours

5.2 Personalization through the avatar and virtual pets

- 1. Personal engagement
