# Supplementary material for: Access to an mHealth Tool for Symptom Management in Pediatric Oncology Care: Triangulation Study
Source: JMIR Form Res. 2026 Jul 2;10:e93934. doi: 10.2196/93934 (PMC13327532; doi:10.2196/93934)
Supplement: Multimedia Appendix 6 [file formative-v10-e93934-s005.docx]

Childrens’ score on SUS-C for each item

| SUS item | SUS statement | n | Md  (min-max) | Number of participants who answered, “strongly agree (4)/ disagree (0)”? |
| --- | --- | --- | --- | --- |
| 1. | I think that I would like to use this system frequently | 14 | 2  (0-4) | 2 participants answered strongly agree. |
| 2. | I found the system unnecessarily complex. | 14 | 0  (0-1) | 12 participants answered strongly disagree. |
| 3. | I thought the system was easy to use. | 14 | 4  (3-4) | 11 participants answered strongly agree. |
| 4 | I think that I would need the support of a technical person to be able to use this system. | 14 | 0  (0-3) | 10 participants answered strongly disagree. |
| 5. | I found the various functions in this system were well integrated. | 12 | 3  (2-4) | 4 participants answered strongly agree. |
| 6. | I thought it was to much inconsistency in this system. | 13 | 0  (0-3) | 7 participants answered strongly disagree. |
| 7. | I would imagine that most people would learn to use this system very quickly. | 14 | 4  (3-4) | 8 participants answered strongly agree. |
| 8. | I found the system very awkward to use. | 13 | 0  (0-2) | 11 participants answered strongly disagree. |
| 9. | I felt very confident using the system | 14 | 3  (2-4) | 6 participants answered strongly agree. |
| 10. | I needed to learn a lot of things before I could get going with this system. | 14 | 0  (0-2) | 10 participants answered strongly disagree. |

Parents’ score for each item

| SUS item | SUS statement | n | Md  (min-max) | Number of participants who answered, “strongly agree (4)/ disagree (0)”? |
| --- | --- | --- | --- | --- |
| 1. | I think that I would like to use this system frequently | 5 | 3  (2-4) | 1 participants answered strongly agree. |
| 2. | I found the system unnecessarily complex. | 5 | 0  (0-2) | 4 participants answered strongly disagree. |
| 3. | I thought the system was easy to use. | 5 | 4  (2-4) | 4 participants answered strongly agree. |
| 4 | I think that I would need the support of a technical person to be able to use this system. | 5 | 0  (0-0) | 5 participants answered strongly disagree. |
| 5. | I found the various functions in this system were well integrated. | 5 | 3  (2-4) | 2 participants answered strongly agree. |
| 6. | I thought it was to much inconsistency in this system. | 5 | 1  (0-2) | 2 participants answered strongly disagree. |
| 7. | I would imagine that most people would learn to use this system very quickly. | 5 | 4  (2-4) | 4 participants answered strongly agree. |
| 8. | I found the system very awkward to use. | 5 | 0  (0-2) | 4 participants answered strongly disagree. |
| 9. | I felt very confident using the system | 5 | 4  (2-4) | 3 participants answered strongly agree. |
| 10. | I needed to learn a lot of things before I could get going with this system. | 5 | 0  (0-2) | 4 participants answered strongly disagree. |

Nurses’ score on SUS-C for each item

| SUS item | SUS statement | n | Md  (min-max) | Number of participants who answered, “strongly agree (4)/ disagree (0)”? |
| --- | --- | --- | --- | --- |
| 1. | I think that I would like to use this system frequently | 6 | 3  (2-4) | 1 participants answered strongly agree. |
| 2. | I found the system unnecessarily complex. | 6 | 0  (0-1) | 5 participants answered strongly disagree. |
| 3. | I thought the system was easy to use. | 6 | 4  (4-4) | 6 participants answered strongly agree. |
| 4 | I think that I would need the support of a technical person to be able to use this system. | 6 | 0  (0-1) | 5 participants answered strongly disagree. |
| 5. | I found the various functions in this system were well integrated. | 6 | 3,5  (2-4) | 3 participants answered strongly agree. |
| 6. | I thought it was to much inconsistency in this system. | 6 | 0  (0-2) | 4 participants answered strongly disagree. |
| 7. | I would imagine that most people would learn to use this system very quickly. | 6 | 4  (3-4) | 5 participants answered strongly agree. |
| 8. | I found the system very awkward to use. | 6 | 0  (0-2) | 5 participants answered strongly disagree. |
| 9. | I felt very confident using the system | 6 | 4  (3-4) | 5 participants answered strongly agree. |
| 10. | I needed to learn a lot of things before I could get going with this system. | 6 | 1  (0-1) | 4 participants answered strongly disagree. |
